# Radiological Screening of Atlantoaxial Instability in Children with Trisomy 21: A Systematic Review and Evidence-Based Recommendations

**DOI:** 10.3390/children12040421

**Published:** 2025-03-27

**Authors:** Leo Cattarinussi, Aline Bregou, Christopher J. Newman, Sophie R. Merckaert

**Affiliations:** 1 Medical School, University of Lausanne, 1011 Lausanne, Switzerland; leo.cattarinussi@unil.ch; 2 Unit of Pediatric Orthopedics and Traumatology, Lausanne University Hospital, 1011 Lausanne, Switzerland; aline.bregou@chuv.ch; 3 Pediatric Neurology and Neurorehabilitation Unit, Lausanne University Hospital, 1011 Lausanne, Switzerland; christopher.newman@chuv.ch

**Keywords:** Down syndrome, Trisomy 21, atlantoaxial instability, cervical spine

## Abstract

**Background/Objectives**: Atlantoaxial instability (AAI) affects approximately 20% of individuals with Trisomy 21. Radiological screening has been debated for decades due to its unclear clinical utility and lack of standardized diagnostic criteria. This systematic review evaluates the indications, efficacy, and clinical implications of radiological screening for AAI in children with Trisomy 21. **Methods**: Following the PRISMA guidelines, we conducted a systematic search in PubMed, Embase, and Google Scholar for studies published between 1990 and May 2024. Studies were included if they assessed AAI screening in pediatric Trisomy 21 populations, defined AAI radiologically, and reported at least two cases. We extracted the demographic data, study design, radiological criteria, screening recommendations, and biases from these studies. **Results**: Of the 537 identified studies, 8 met the inclusion criteria, encompassing 2536 children (mean age: 7 years). Five studies supported routine screening, while three opposed it. Studies varied significantly in their AAI definitions, using atlanto-dental interval (ADI) thresholds of 4 mm to 6 mm, the space available for cord (SAC), and the basion-axial interval (BAI). No study demonstrated a definitive correlation between radiological findings and neurological symptoms. **Conclusions**: Routine radiological screening for AAI in asymptomatic children with Trisomy 21 is not supported by consistent evidence. A selective screening approach, focusing on symptomatic patients or those engaging in high-risk activities, may be more appropriate. The standardization of radiological criteria and prospective studies are needed to refine screening recommendations.

## 1. Introduction

Trisomy 21 is the most common chromosomal anomaly, with an estimated incidence of about 1 in 1000 births [1]. The clinical presentation of Trisomy 21 is multisystemic, with common features including hypotonia; joint laxity; a distinctive facial phenotype; congenital heart defects; sleep apnea; hematological disorders such as leukemia; epilepsy; thyroid dysfunction; early-onset Alzheimer’s disease, which typically occurs around the age of 40; as well as neurodevelopmental issues, such as speech and language disorders and autism spectrum disorders [2,3,4]. Musculoskeletal anomalies in Trisomy 21 are frequent and include a short stature, increased fracture risk, joint laxity, developmental hip dysplasia, and skeletal malformations [5]. Atlantoaxial instability (AAI) is one of the most frequent musculoskeletal features, occurring in approximately 20% of individuals. It arises from C1-C2 joint laxity and is often associated with a C2 malformation known as os odontoideum (OO) [6,7].

The atlantoaxial joint is considered to be unstable when there is excessive motion between the C1 and C2 vertebrae, increasing the risk of dislocation and potential acute or chronic spinal cord injuries.

Diagnosing AAI in Trisomy 21 is challenging due to the absence of specific clinical signs and the risks associated with cervical spine manipulation. Suspicion of symptomatic instability is established on indirect clinical signs during follow up, such as pyramidal neurological manifestations and/or motor skill regression. Radiological evaluation has been utilized for decades in the assessment of AAI, initially focusing on lateral cervical spine radiographs to measure the atlanto-dental interval (ADI) and the space available for cord (SAC) [8]. Historically, routine radiological screening was recommended for all children with Trisomy 21 to detect AAI before neurological symptoms appeared. However, this practice was challenged in 2011 when the American Academy of Pediatrics (AAP) withdrew its recommendation, citing insufficient evidence supporting the clinical utility of routine screening [9].

Despite this, some researchers still advocate for imaging in specific cases where clinical suspicion is high. However, there is no consensus on the accuracy of radiological screening and concerns persist regarding overdiagnosis and the lack of a standardized radiological threshold for the diagnosis of AAI, or for the preferred imaging technique.

This systematic literature review aims to evaluate the indications and efficacy of radiological screening for AAI in children with Trisomy 21, to analyze the variability in screening criteria, as well to propose evidence-based recommendations for screening protocols in children with Trisomy 21.

## 2. Materials and Methods

### 2.1. Search Strategy and Selection Criteria

In accordance with the ‘Preferred Reporting Items for Systematic Reviews and Meta-Analyses’ (PRISMA-P) statement, we conducted a comprehensive search of the PubMed, Embase, and Google Scholar bibliographic databases to identify all relevant studies published between 1990 and May 2024. Keywords and index terms (MeSH headings) related to Trisomy 21 and AAI were used. The language was restricted to English, French, and Italian [10].

The full search strategy is summarized in Table 1.

### 2.2. Study Selection

Studies were eligible for inclusion if they fulfilled the following criteria:

- The study explicitly discussed whether screening was recommended.
- The study clearly defined the radiological definition of AAI.
- The study reported at least 2 cases of children with AAI. This threshold was chosen to ensure that studies included a minimum level of patient data for meaningful analysis, reducing bias from isolated case reports.
- The study only included participants under 25 years old, with a mean age under 18.
- The study only included participants with a confirmed diagnosis of Trisomy 21.

Meta-analyses, literature reviews, non-peer-reviewed studies, and studies published before 1990 were excluded.

### 2.3. Data Collection Process

Titles and abstracts were screened by two reviewers (LC and SM), with full-text evaluation performed for eligible studies. Discrepancies were resolved by a third reviewer (AB).

For each full-text article that was analyzed, the following variables were evaluated:

- Study design (case reports, case–control, double-blind studies, prospective or retrospective studies);
- Demographic data (age, biological sex);
- Definition of AAI used by the authors;
- Identified biases;
- Screening implementation (yes or no);
- Types of screening methods.

The frequency and percentage were used for the categorical data while the mean and range were used for the continuous data. Due to the study heterogeneity, a meta-analysis was not performed.

## 3. Results

**Figure 1:**
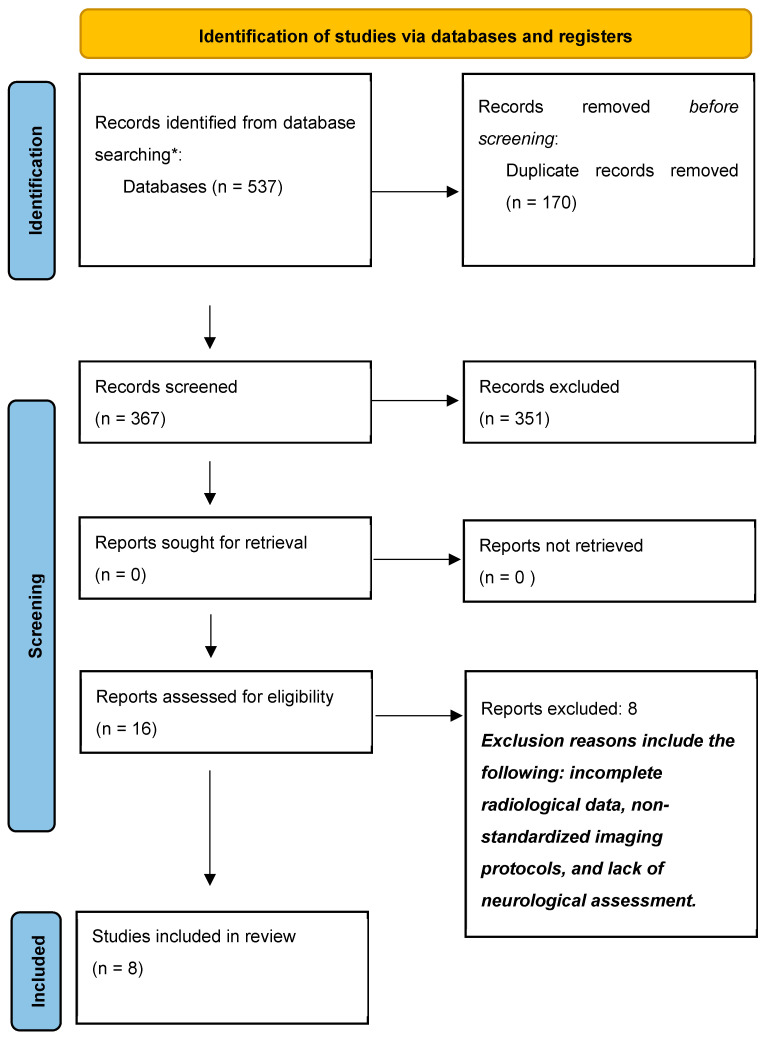
Flowchart of the study selection process according to PRISMA. * Medline, Embase, google scholar.

Our search strategy retrieved a total of 537 articles and 16 publications were selected for full-text analysis based on their title and abstract. According to our selection criteria, eight articles were included in our review, including 2536 cases (Figure 1).

Among the selected articles, two pairs of studies were authored by the same researchers, potentially leading to duplicate data [8,11,12,13]. The mean participant age was 7 years (range: 4 months to 24 years). Three studies did not report the mean age [14,15,16]. Regarding biological sex, 58% were male and 42% were female, with four studies lacking sex data [8,14,15,17]. The key findings are summarized in Table 2.

Six studies were retrospective [8,11,12,13,14,15], and two were prospective [16,17] (Table 2).

All were conducted in a single center.

Five studies supported radiological screening [8,11,12,13,15], while three opposed it, reporting a lack of correlation between radiological findings and neurological symptoms [14,16,17].

The radiological criteria used were the atlanto-dental interval (ADI) with thresholds varying between 4 mm and 6 mm; the space available for cord (SAC) with a threshold < 14 mm; the basion-axial interval (BAI), which was only included in two studies, and finally, the C1 inclination and SAC ratio proposed by Nakamura et al. were used as alternative markers [11,12].

Two studies presented significant limitations and biases [12,15]. In the study by Morton et al., the authors did not report the demographic data of their patients and the distribution of ADI measurements. Furthermore, the methodology for measuring ADI differed between the two comparison groups. Selby et al. attempted to find a correlation between abnormal neurological exams and increased ADI measurements on radiographs, but none of their participants had a real neurological deficit, making the results difficult to interpret [17].

Hengartner et al. [14] provided the largest dataset, representing 80% of the Trisomy 21 population under 18 in their region. Their findings suggested that discontinuing systematic screening did not increase adverse outcomes.

## 4. Discussion

To our knowledge, this literature review is the first to rigorously apply inclusion and exclusion criteria based on the PRISMA-P model for AAI screening in children with Trisomy 21.

Routine screening for AAI in patients with Trisomy 21 remains strongly debated. The reviewed studies provided a range of perspectives on the prevalence, radiological definitions, and implementation of screening protocols for AAI.

While some studies advocated for early radiological screening at 3–5 years old to detect potential instability, others raised concerns about the clinical relevance of such findings and the risks associated with overdiagnosis.

Numerous studies emphasized the high prevalence of AAI in young children with Trisomy 21 and suggested that early radiological screening could enable the early detection of at-risk cases before clinical symptoms manifested. For example, Bouchard et al. and Bauer et al. supported the idea that radiological screening may identify AAI early, potentially before any neurological deficits develop [8,13]. These studies typically recommended screening at 3 years of age, using parameters such as an ADI greater than 6 mm or an SAC less than 14 mm. Early detection, in this context, is viewed as a safeguard against severe neurological damage, which may be more easily prevented if identified prior to the onset of clinical symptoms.

Conversely, researchers such as Selby et al. and Cremers et al. presented an alternative perspective, questioning the clinical significance of the radiological findings of AAI [16,17]. These studies reported that many individuals with radiological instability did not experience neurological deficits, thereby suggesting that the presence of radiological AAI does not necessarily equate to a clinically significant condition. Selby et al. [17] notably found no neurological impairments in patients who showed clear signs of AAI, raising concerns that the detection of radiological instability may lead to overdiagnosis. This, in turn, may result in unnecessary interventions, such as restrictions on physical activity, which could generate undue anxiety for families and cause an unnecessary burden on both patients and caregivers.

A further point of discussion is whether the studies implemented radiological screening as part of their study protocol (a priori) or whether they retrospectively evaluated cases and then formulated recommendations (a posteriori). In our review, several studies employed a priori screening protocols. These studies used specific radiological methods—such as lateral cervical spine X-rays in flexion or in neutral positions at predetermined ages—and, based on their findings, subsequently recommended routine screening [8,11,12,13,15].

In contrast, other authors did not implement a screening protocol; instead, they retrospectively analyzed radiographic data and found no clear neurological deficits, leading them to question the utility of routine screening [16,17].

### 4.1. Biases and Limitations

The radiological criteria used varied across studies, including the atlanto-dental interval (ADI), with thresholds ranging from 4 mm to 6 mm [8,13,15,16]; the space available for the cord (SAC), with a threshold of <14 mm; and the basion-axial interval (BAI), which was assessed in only two studies. Additionally, Nakamura et al. proposed alternative markers, such as C1 inclination and the SAC ratio, to improve diagnostic accuracy [11,12].

Studies like Morton et al. suggested screening with lateral cervical spine X-rays in flexion positions, while others favored neutral positions [8,11,15]. Flexion X-rays may be more sensitive for detecting instability but can be technically difficult and expose patients to additional radiation.

Furthermore, Morton et al. demonstrated that when two radiographs are taken just 10 min apart, there can be up to a 1 mm difference in the ADI measurement in 37% of patients. This highlights the inherent imprecision in using ADI as the sole diagnostic criterion, reinforcing the need for a more comprehensive approach that considers multiple radiological parameters [8,11,12,15,16,17,18]. 

Variations in radiological criteria significantly impact clinical decision-making, diagnosis, and treatment planning. The reliance on multiple diagnostic parameters rather than a single standardized measure represents a major limitation. Indeed, the lack of consensus on radiological thresholds leads to inconsistencies in identifying at-risk patients, which may result in the overdiagnosis or underdiagnosis of AAI in children with Trisomy 21. The differences in defining ADI, SAC, and other parameters directly influence whether a child is classified as having instability, impacting recommendations for physical activity, surgical intervention, and follow-up strategies. For example, studies utilizing an ADI threshold of 4 mm may identify more cases of AAI compared to those using a 6 mm cutoff, leading to varying treatment decisions. Similarly, the use of flexion vs. neutral lateral X-rays introduces discrepancies in screening outcomes, with some clinicians favoring one method over the other. These inconsistencies highlight the urgent need for standardized screening protocols to ensure uniform patient care and minimize unnecessary interventions.

Retrospective studies may suffer from incomplete data or selection bias [11,12,15].

Additionally, the wide variability in age ranges in several studies complicated the interpretation of results, since AAI may develop or resolve over time [8,16]. Younger children are more likely to have radiological instability due to ligamentous laxity, which may not persist into adolescence or adulthood.

It is also important to consider the inherent challenges in obtaining cervical radiographs in children with Down syndrome, particularly in those who may not fully understand the procedure and have difficulty remaining still for long periods. This can compromise the accuracy of the radiographic measurements.

### 4.2. Clinical Implications

A key argument against routine screening is the low rate of symptomatic AAI in Trisomy 21 patients, estimated to be less than 1% [19]. Selby et al. and Cremers et al. reported no neurological deficits, suggesting that many radiological findings are clinically insignificant [16,17]. Implementing routine screening may lead to false positives, causing undue stress and potential overtreatment, even though some authors, such as Nakamura et al., have proposed more advanced radiological parameters to improve the accuracy of screening and to identify patients at higher risk [11,12]. Additionally, Hengartner et al. [14] provided robust data, encompassing approximately 80% of the Down syndrome pediatric population in their region. Their findings imply that discontinuing routine radiological screening—aligned with the American Academy of Pediatrics’ recommendation from 2011 against screening asymptomatic patients—has not resulted in a significant increase in accidents or surgical interventions related to atlantoaxial instability [14].

Furthermore, it is particularly striking that none of the analyzed studies address the psychological impact of an AAI diagnosis on patients or their families. The incidental identification of AAI on an asymptomatic X-ray has the potential to lead to substantial anxiety among both patients and their caregivers. The associated burden of frequent medical appointments, invasive imaging procedures such as MRIs, and the restrictions on physical activity may exacerbate psychological distress. This can add extra stress for caregivers, who already have a lot to manage when caring for children with Trisomy 21.

## 5. Conclusions

Based on the reviewed literature, routine radiological screening for AAI in all children with Trisomy 21 is not warranted. Instead, a strategy of selective screening should be considered for patients presenting with neurological symptoms, gait abnormalities, or who engage in high-risk activities (e.g., contact sports). Future research should aim to standardize the diagnostic criteria for AAI and identify Trisomy 21 subpopulations at the highest risk of developing symptomatic AAI.

## Figures and Tables

**Table 1:** Summary of keywords and index terms (MeSH headings) [10].

Database	Code	Filter	Date	Found
Medline (Pubmed)	(“Down Syndrome”[MeSH] OR “down syndrome”[tiab] OR Mongolism OR “47,XY,+21”[tiab] OR “Trisomy G”[tiab] OR “47,XX,+21”[tiab] OR “Down’s Syndrome”[tiab] OR “Downs Syndrome”[tiab] OR Trisomy 21[tiab]) AND (“Atlanto-Axial Joint”(5) OR (“Cervical Vertebrae”(5) AND “Joint Instability”[Mesh]) OR Atlanto-Axial[tiab] OR Atlantoaxial[tiab] OR AAI[tiab])	Child: Birth–18 years Result by year: 1990–2024	2 May 2024	161
Embase	(‘Down syndrome’/exp OR ((down* NEAR/3 (syndrome* OR disease OR langdon)) OR (trisomy* NEAR/1 (g OR 21)) OR “47,X?,+21”):ab,ti,kw) AND (‘atlantoaxial joint’/exp OR ‘atlantoaxial dislocation’/exp OR (‘cervical vertebra’/exp AND ‘joint instability’/de) OR (Atlanto-Axial OR Atlantoaxial OR AAI OR (atlas NEAR/3 (disloca* OR luxat* OR instab*))):ab,ti,kw) NOT (‘adult’/exp NOT (‘child’/exp OR ‘adolescent’/exp))	Publication year: 1990–2024	2 May 2024	276
Google Scholar	Down|trisomy atlantoaxial|atlanto-axial	The first 100 suggestions	2 May 2024	100
Total				537

**Table 2:** Key findings of the eight selected studies.

Author (Year)	Study Design	Included Patients	Mean Age (Years) (Range)	Sex Ratio (M/F)	Radiological Definition of AAI	Biases	Screening Implementation (Yes or No)	Type of Screening Method
Selby et al. (1991) [17]	Observational prospective study	135	9.3 (6–14)	Not reported	ADI ≥ 4.5 mm	No patient with clear neurological deficit.	No	Not reported
Cremers et al. (1993) [16]	Cohort prospective study	91	Not reported (4–20)	65/26	ADI ≥ 4 mm	None	No	Not reported
Morton et al. (1995) [15]	Cohort retrospective study	90	Not reported (4–19)	Not reported	ADI ≥ 4 mm	Average age unspecified, distribution of ADI measures unspecified.	Yes	Lateral cervical spine X-ray in flexion position at 4–5 years old
Nakamura et al. (2014) [11]	Case–control retrospective study	50	3.1 (0.4–17)	24/26	1/4SAC ^1^ ratio < 0.86, C1 inclination ^2^ >10°	None	Yes	Lateral cervical spine X-ray in neutral position
Nakamura et al. (2016) [12]	Case–control retrospective study	272	5.5 (2–12)	156/116	1/4SAC ^1^ ratio < 0.86, C1 inclination ^2^ >10°	None	Yes	Lateral cervical spine X-ray in neutral position
Bouchard et al. (2019) [8]	Observational retrospective study	172	8.33 (0–25)	Not reported	ADI > 6 mm, SAC < 14 m, BAI > 12 mm	None	Yes	Lateral cervical spine X-ray in neutral position at 3 years old
Hengartner et al. (2020) [14]	Observational retrospective study	1566	Not reported	Not reported	ADI > 4.5 mm	None	No	Not reported
Bauer et al. (2021) [13]	Observational retrospective study	160	7.4 (3–20.8)	88/72	ADI > 6 mm, SAC < 14 mm, BAI > 12 mm	None	Yes	Lateral cervical spine X-ray in neutral position at 3 years old

^1^**1/4SAC**: “the ratio of the anteroposterior diameter of the spinal canal at the level of C1 to that at the level of C4” [11]. ^2^ **C1 inclination**: “the angle formed between the line perpendicular to the tangent at the posterior surface of the body of C2 and the line connecting the centres of the anterior and posterior arches of C1” [11].

## Data Availability

Data are available on request. The data are not publicly available due to technical and time limitations.

## Abbreviations

The following abbreviations are used in this manuscript:

AAI	Atlantoaxial instability
OO	Os odontoidum
SAC	Space available for cord
ADI	Atlanto-dental interval
BAI	Basion-axial interval
